# Notes of Hospital Practice

**Published:** 1872-11

**Authors:** S. D. Gross

**Affiliations:** Jefferson Medical College—Clinic


### NOTES OF HOSPITAL PRACTICE.

### Jefferson Medical College—Clinic of Prof. S. D. Gross, M. D.

STRUMOUS HYPERTROPHY OF THS LIP.

This case, a boy ten years old, exhibited a peculiar development of the upper lip, which was thickened and pendulous. This condition had existed for three years; otherwise he enjoyed good health.

This is a curious affection, but it is one that is occasionally met with in young persons with the strumous diathesis. It occurs almost exclusively in the upper lip and in the young of both sexes. In these cases the lip has twice its natural thickness, is hard and rigid, and sometimes shows a tendency to ulcerate. The affection is frequently accompanied by strumous manifestations in other portions of the system, such as enlarged tonsils and psorophthalmia. The firmness and induration of the lip, the absence of co-existing disease in the gums and teeth, and the chronic nature of the enlargement, together with the scrofulous appearance and condition of the patient, will generally make the diagnosis sufficiently clear. No operation is needed for its relief. Attention to the secretions, with some alterative medicine, or a mild course of mercury when other means fail, will generally be all the treatment required.

The patient has a broad nose, and his face has a heavy expression, as if his mental operations were slow. He states that there is no disease in the family, of which he is aware, and his parents are healthy people. His tongue is a little coated, but no more than the American tongue generally is, because everybody eats too much in this country. We find no enlarged glands along the sterno-cleido-mastoid or under the angle of the jaw, which so frequently exists in scrofulous subjects. If they were here, however, we should not bring that fact forward as conclusive proof that this, or any disease here, was scrofulous, as they might be enlarged independent of any disease. But from other sources we derive the evidence that this is an enlargment of the lip of strumous origin. We may distinguish two kinds of scrofulous subjects. The first variety are blondes, with blue eyes and a light florid complexion, with a vigorous circulation and a bright precocious mind. In these the disease manifests itself in the bones, joints, skin, and lymphatic glands, and they are not so liable to suffer from consumption as the second class, who are generally dark, with a pale und unhealthy complexion, tumid lip, and dropping eyelashes; they are dull and languishing, and show no activity of brain or muscle. When I was in Louisville I attended a young lady of nineteen years, who was a blonde with very light hair. Though in other respects healthy, her upper lip was immensely enlarged and indurated to the touch, more so than in the present case. In the case referred to, I derived great benefit from the officinal solution of baryta. This is a powerful detergent and alterative, and is especially adapted to strumous cases where there is a deficiency of blood, and the face and lips are pallid; this is not exactly the case here, but it will make a decided improvement in the boys appearance in a short time.

R. Liquor. barytæ, gtt. iv.

Tinct. ferri chlorid., gtt. x. M.

S. To be taken in a tablespoonful of water, morning, after dinner, and at bedtime.

We will make no change in his diet; he may take his accustomed food, taking care, however, that he has out-door exercise, and that his bowels are kept in a soluble condition. We will see him from time to time, and note his progress.—*Med. Times.*

